# Total flavones of *Abelmoschus manihot* improve diabetic nephropathy by inhibiting the iRhom2/TACE signalling pathway activity in rats

**DOI:** 10.1080/13880209.2017.1412467

**Published:** 2017-12-08

**Authors:** Su Liu, Lifang Ye, Jing Tao, Chao Ge, Liji Huang, Jiangyi Yu

**Affiliations:** a Department of Endocrinology, Jiangsu Province Hosipital of TCM, Affiliated Hospital of Nanjing University of Chinese Medicine, Nanjing, China;; b Department of Nephrology, Jiangsu Province Hosipital of TCM, Affiliated Hospital of Nanjing University of Chinese Medicine, Nanjing, China;; c Department of Gastroenterology, Jiangsu Province Hosipital of TCM, Affiliated Hospital of Nanjing University of Chinese Medicine, Nanjing, China

**Keywords:** TFA, endoplasmic reticulum stress, renal injury, AGEs

## Abstract

**Context:** Total flavones extracted from *Abelmoschus manihot* L. (Malvaceae) medic (TFA) have been proven clinically effective at improving renal inflammation and glomerular injury in chronic kidney disease (CKD).

**Objective:** This study evaluated the function of TFA as an inhibitor of iRhom2/TACE (tumour necrosis factor-α converting enzyme) signalling and investigated its anti-DN (diabetic nephropathy) effects in a DN rat model.

**Materials and methods:***In vitro*, cells were treated with 200 μg/mL advanced glycation end products (AGEs), and then co-cultured with 20 μg/mL TFA for 24 h. Real time PCR, western blotting and co-immunoprecipitation assays were performed. *In vivo*, DN was induced in 8 week old male Sprague-Dawley rats via unilateral nephrectomy and intraperitoneal injection of streptozotocin, then TFA were administered to rats by gavage for 12 weeks at three different doses (300, 135 and 75 mg/kg/d). 4-Phenylbutanoic acid (2.5 mg/kg/d) was used as a positive control.

**Results:** IC_50_ of TFA is 35.6 μM in HK2 and 39.6 μM in HRMC. TFA treatment (20 μM) inhibited the activation of iRhom2/TACE signalling in cultured cells induced by AGEs. LD_50_>26 g/kg and ED_50_=67 mg/kg of TFA in rat by gavage, TFA dose-dependently downregulated the expression of proinflammatory cytokines and exerted anti-inflammatory effects significantly though inhibiting the activation of iRhom2/TACE signalling.

**Discussion and conclusions:** Our results show that TFA could dose-dependently ameliorate renal inflammation by inhibiting the activation of iRhom2/TACE signalling and attenuating ER stress. These results suggest that TFA has potential therapeutic value for the treatment of DN in humans.

## Introduction

Diabetic nephropathy (DN) represents one of the most important health problems worldwide (Danaei et al. 2011; Zimmet et al. 2014). Approximately, one-third of all diabetic patients are affected by DN (Atkins and Zimmet 2010), which constitutes the most frequent cause of end-stage renal disease (Balakumar et al. 2012; Lv et al. 2015). DN shows progressive renal damage over several years and involves characteristic pathological changes such as the accumulation of extracellular matrix (ECM), glomerulosclerosis and interstitial fibrosis (Duran-Perez et al. 2011). DN is one of the leading causes of chronic renal failure (Kerner et al. 2014), resulting in significant social and economic burdens (Cooper 2012).

Currently, strict control of major modifiable risks such as hypertension, glucose levels and dyslipidaemia is the primary therapeutic strategy for DN (Donate-Correa et al. 2015). However, there are no effective ways to prevent the development of DN in diabetic patients, for the molecular events of DN remain incompletely understood. Therefore, the identification of therapies that specifically target DN by affecting the primary mechanisms that contribute to its pathogenesis is desperately needed. Such treatments in combination with reduction of risk factors through lifestyle changes could provide significant clinical benefit to diabetic patients (Williams and Tuttle 2005). There are convincing data that relate the diabetes inflammatory component with the development of renal disease (Donate-Correa et al. 2015). Diabetic nephropathy is considered an inflammatory disease, and several reports recently demonstrated inflammasome activation in association with DN (Shahzad et al. 2015). The inflammation process underlies the mechanisms of DN progression. Therefore, anti-inflammatory strategies may offer approaches of great interest in DN therapy. Diverse *in vitro* and *in vivo* studies have shown that angiotensin-converting enzyme inhibitors or other therapeutic agents such as pentoxifylline (PTF) are able to reduce the levels of the main proinflammatory cytokines that are related to renal hypertrophy and increased urinary albumin (Alb) excretion (Donate-Correa et al. 2015). However, further clinical trials are necessary to examine the potential renal protective effects of the above therapeutic agents of DN. As the modulation of inflammatory processes might be useful in the prevention or therapy of DN, understanding the regulation of inflammatory pathways in this clinical complication will provide new therapeutic targets.

The cytokine tumour necrosis factor (TNF) is the primary trigger of inflammation and is associated with many human diseases including rheumatoid arthritis, Crohn’s disease, atherosclerosis, psoriasis, sepsis, diabetes and obesity (Adrain et al. 2012). Its blockade is considered as a therapy for a number of diseases and is being assessed for others (Tracey et al. 2008). TNF signalling requires iRhom2 to promote trafficking and activation of tumour necrosis factor-α converting enzyme (TACE) (Adrain et al. 2012). iRhoms are non-protease members of the rhomboid-like super family of polytopic membrane proteins. They are endoplasmic reticulum (ER) proteins that regulate the trafficking and fate of membrane proteins in a variety of contexts (Zettl et al. 2011; Adrain et al. 2012; McIlwain et al. 2012). Mouse iRhom2 is one of the best understood members of the iRhom family. It is a myeloid-specific regulator of the activation of TACE, an important shedding protease that releases proteins from the cell surface (Gooz 2010). Tumour necrosis factor-α converting enzyme (also called ADAM17) was first discovered as the enzyme responsible for the release of active TNF (Peschon et al. 1998). IRhom2 binds TACE and promotes its exit from the ER, which is required for TNFα release in mice (Adrain et al. 2012). The failure of TACE to exit the ER in the absence of iRhom2 prevents TNFα mature and secretion. Thus, given the role of TNFα in inflammatory diseases, iRhom2 may represent an attractive therapeutic target for DN (Adrain et al. 2012).

Many traditional Chinese medicines are commonly used for the treatment of inflammatory diseases and other ailments. *Abelmoschus manihot* L. (Malvaceae) medic (AM), is a traditional Chinese medicine widely used to treat inflammatory diseases (Tu et al. 2013). The total flavones of *A. manihot* (TFA) are extracted from AM and have been approved by the China State Food and Drug Administration (Z19990040) for the treatment of nephritis. The main bioactive components of TFA including isoquercitrin (C21N20O12), hibifolin (C21N18O14), myricetin (C15N10O8), quercetin-3′-*O*-d-glucoside (C21N20O12), quercetin (C15N10O7), hyperoside (C21N20O12) and gossypetin (C15N10O8) have been standardized by high performance liquid chromatography (HPLC) (Trendafilova et al. 2011; Xue et al. 2011). It has been reported that TFA improve renal inflammation, nephrotic syndrome, purpura nephritis, IgA nephropathy, membranous nephropathy and DN effectively in the clinical trial (Liu et al. 2012; Tu et al. 2013; Ge et al. 2016). However, the molecular mechanisms by which TFA elicit an anti-inflammatory effect as well as its pharmacological mechanisms in treating DN remain unclear.

In this study, we examined the effects and mechanism of TFA on the activation of the iRhom2/TACE signalling pathway *in vitro* and *in vivo*. We also examined the protective effects of TFA on DN using a streptozotocin-induced DN rat model.

## Materials and methods

### TFA

TFA were extracted and characterized from flowers of *A. manihot* by the Department of Chinese Materia Medica, Nanjing University of Chinese Medicine (Nanjing, China) from August 2015 to December 2015. The extraction process of TFA are as follows: three extractions with 70% alcohol for 50 min each, and the yield was about 35% (Lai et al. 2007; Tu et al. 2013). TFA are mainly composed of seven flavone glycosides, which were characterized by HPLC, and their chemical structures were identified. The TFA profile composition was the following: 43.2% hyperoside, 27.1% hibifolin, 13.7% isoquercetin, 8.8% quercetin-3′-O-glucoside, 3.8% quercetin-3-*O*-robinobioside, 3.2% myricetin and 0.2% quercetin (Trendafilova et al. 2011; Xue et al. 2011).

### Cell culture and treatments

A human proximal tubule epithelial cell line (HK-2) and human renal mesangial cells (HRMC) were obtained from the American Type Culture Collection (ATCC, Manassas, VA). HK-2 was cultured in DMEM/F-12 medium (GIBCO, 11330-057, Carlsbad, CA). HRMC was cultured in complete growth medium DMEM (Hyclone, Logan, UT) that was supplemented with 10% foetal bovine serum (GIBCO, Carlsbad, CA), 100 U/mL penicillin and 100 μg/mL streptomycin at 37 °C in a humidified atmosphere of 5% CO_2_ (Invitrogen, Carlsbad, CA).

HK-2 and HRMC were grown for 3–5 passages to approximately 70% confluence. For advanced glycation end products (AGEs)-stimulation experiments, the cells were serum-starved in medium containing 0.1% serum and 5.5 mM glucose. After 24 h, the medium was replaced with fresh medium containing 5.5 mM glucose and 200 μg/mL AGEs or only 5.5 mM glucose, and then the cells were treated with 20 μg/mL TFA or vehicle. AGEs were prepared *in vitro*, as previously described (Li et al. 2011).

### RNA interference ablation of iRhom2

Small interfering RNA (siRNA) specific to iRhom2 was designed (the sequence is listed in Supplementary Table 1), synthesized, and used to ablate iRhom2 in HK-2. Briefly, HK-2 was grown in DMEM/F12 supplemented with 10% FBS, washed with Opti-MEM and incubated for 60 min with Opti-MEM media before transfection with siRNA oligonucleotides. The cells were incubated with 1 μM iRhom2 siRNA or scrambled control oligonucleotides using Lipofectamine 2000 (Invitrogen, Carlsbad, CA) as the transfection reagent. After 12 h, the medium was replaced with fresh DMEM/F12 (containing 10% FBS), and the samples were incubated for another 12 h. Using this procedure, an HK-2 transfection efficacy of approximately 70% was obtained, and the levels of iRhom2 protein were reduced by more than 60%.

### RT-PCR

Total RNA was extracted using TRIzol reagent (Invitrogen, Carlsbad, CA) and subjected to reverse transcription using reverse transcriptase (TAKARA, Dalian, China). Samples were amplified by PCR using gene-specific primers (listed in Supplementary Table 1), and the StepOnePlus™ Real-Time PCR System (Life Technologies, Camarillo, CA).

### Western blot analysis

Protein lysates were resolved using 12% sodium dodecyl sulphate polyacrylamide gel electrophoresis (SDS-PAGE) and transferred to PVDF membranes (Millipore, Bedford, MA). Membranes were blocked with 5% skim milk for 1 h, followed by incubation with primary antibodies (Santa Cruz Biotechnology Inc., Santa Cruz, CA). Immune complexes were detected using the enhanced chemiluminescence detection system (Tanon, Shanghai, China).

### Co-immunoprecipitation

For immunoprecipitation analysis, HK-2 cell lysates (3 mg protein) were mixed with the indicated antibody (2 μg) at 4 °C overnight, followed by the addition of 80 μL of protein G-Sepharose (Beyotime, Nantong, China) for 1.5 h at 4 °C. Immune complexes were washed five times with lysis buffer (Cell Lysis Buffer from Cell Signaling, Foster City, CA) that was supplemented with complete mini-protease inhibitor cocktail (Roche Applied Science, Indianapolis, IN). After boiling in 5× sample buffer, samples were subjected to SDS/PAGE, transferred to nitrocellulose using the iBlot Dry Blotting System (Invitrogen, Carlsbad, CA), and then immunoblotted with the indicated primary antibodies.

### Animals

Adult male Sprague-Dawley rats weighing from 170 to 215 g were purchased from the Animal Center of Nanjing Military District General Hospital (Nanjing, China). Rats were treated according to the guidelines of the Animal Ethics Committee of Nanjing University Medical School. Animals were housed at constant room temperature (21 °C) under a controlled 12 h light/dark cycle and had free access to water and standard laboratory diet. The surgical procedures and experimental protocol were approved by the Animal Ethics Committee of Nanjing University of Traditional Chinese Medicine.

### Streptozotocin-induced DN and drug treatment

One hundred healthy 8 week old male Sprague-Dawley rats were purchased. The DN rat model was established by unilateral nephrectomy and induced by a single intraperitoneal injection of streptozotocin (Sigma Aldrich, St. Louis, MI) at 35 mg/kg body weight (BW) in 0.01 M citrate buffer (pH 4.2) after a 16 h overnight fast (Sun et al. 2013). Body weight, water intake, urine volume, urinary Alb, 24 h urinary protein content, blood glucose level, glycosylated haemoglobin, blood lipid, serum creatinine (Scr) and urea nitrogen were measured, and renal pathology was examined to evaluate the success and stability of the rat model (Ozcan et al. 2012). The rats were randomly allocated into the following experimental groups: normal control rats (non-DN group, *n* = 20), streptozotocin-induced DN rats (DN group, *n* = 20), high-dose TFA-treated DN rats (DN group treatment +300 mg TFA/kg/d group, *n* = 20), middle-dose TFA-treated DN rats (DN group treatment +135 mg TFA/kg/d group, *n* = 20), low-dose TFA-treated DN rats (DN group treatment +75 mg TFA/kg/d group, *n* = 20) and 4-phenylbutanoic acid (PBA)-treated DN rats (DN group treatment +2.5 mg PBA/kg/d group, *n* = 20). TFA was dissolved in distilled water to a concentration of 0.5 g/mL for experimental use and was called the AM suspension. TFA was administered to rats by gavage for 12 weeks (Tu et al. 2013). As a positive control, PBA (Sigma Aldrich, St. Louis, MI) was administered at a dose of 2.5 mg/kg BW by gavage for 12 weeks.

### Physiological and metabolic parameters

The behaviour, drinking water, diet, fur colour, and activities of the rats were observed daily (Tu et al. 2013). The rats were weighed every weekend before and after the administration of drugs or distilled water. The kidney index was determined as 1000 × kidney weight (KW)/BW (Sun et al. 2013). A 24 h urine collection was obtained using metabolic cages. Urinary protein concentrations were determined with Coomassie Brilliant Blue as described previously (Tu et al. 2013). At the end of the 12th week after administration, the rats were anesthetized with ketamine, the chests were carefully incised, and 5 mL blood was drawn from the heart. Serum Alb, Scr, serum blood urea nitrogen (BUN), serum triglycerides and HDL cholesterol levels were determined using an automatic biochemical analyser in the Department of Laboratory Medicine of Nanjing Drum Tower Hospital (Tu et al. 2013). Serum adiponectin was determined by rat enzyme-linked immunosorbent assay (ELISA) kits (R&D Systems, Minneapolis, MN). TNF-α, interleukin (IL)-6 and MCP-1 levels were determined by real-time PCR and ELISA. Glomerulosclerosis is defined as the accumulation of ECM deposits and mesangial expansion (Cignarella et al. 2006). The glomerulosclerosis index (GSI) was assessed in periodic acid-Schiff-stained sections in 75 randomly selected glomeruli, and the degree of sclerosis was graded using a semi-quantitative scoring method (Saito et al. 1987).

### Immunohistochemistry

Paraformaldehyde-fixed kidney sections (4 μm thick) were mounted on slides, dewaxed and hydrated. Slides were boiled in 10 mM sodium citrate buffer (pH 6) for 2 min and cooled on bench top for 30 min. After 15 min incubation in 3% hydrogen peroxide, sections were blocked with normal goat serum for 30 min and then incubated with primary antibodies for overnight at 4 °C. After washing with PBST buffer for three times, sections were incubated with horseradish peroxidase-conjugated anti-rabbit or anti-mouse IgG for 30 min. Localization of peroxidase conjugates was determined using diaminobenzidine tetrahydrochloride solution as a chromogen and haematoxylin for counterstaining.

### Measurement of TACE activity

TACE activity in lysates of HK-2 or renal tissues was measured using a fluorescence-quenching substrate for ADAM17/TNF-α (Peptide International, Louisville, KY) as described earlier (Jin et al. 2002). Briefly, HK2 or HRMC was treated with AGEs or AGEs plus TFA for 24 h. The cells were then collected in activity buffer containing 50 mM Tris–HCl (pH 7.4), 25 mM ZnCl_2_, 4% glycerol and 0.5% NP-40. The cells were lysed by freeze–thaw, and the supernatants were collected. Equal amounts of cell lysates were incubated with 5 μM TACE substrate for 30 min at 37 °C. Changes in fluorescence were monitored at excitation wavelength (ex) 320 nm and emission wavelength (em) 420 nm using a fluorescence plate reader. Fluorescence quenching was used to calculate percent activity using appropriate control and blank values.

### ROS production

ROS production in renal tissues was measured in frozen kidney sections embedded in Optimal Cutting Temperature embedding medium. Sections were covered with 10 μmol/L 2′,7′-dichlorofluorescein (DCF) diacetate and incubated in the dark at 37 °C for 30 min. Slides were washed with PBS for 5 min and mounted with VectaShield Mounting Mediafor Fluorescence (Vector Laboratories, Burlingame, CA). Images were acquired with an Olympus microscope (Olympus, Tokyo, Japan) under fluorescent light. Quantification was made as pixel density from 30 random images per group using MetaMorph software (Molecular Devices Corp., Sunnyvale, CA). ROS production in HK2 was measured as previously described (Zhu et al. 2011).

### Statistical analysis

All data were expressed as means ± SD. One-way ANOVA was used to determine statistically significant differences between groups. Scheffe’s test (SPSS12.0; SPSS, Chicago, IL) was used to correct for multiple comparisons when statistical significances were identified in the ANOVA. Statistical significance was set at *p* < 0.05.

## Results

### The expression of iRhom2/TACE in HRMC and HK-2 cell lines

**Figure 1.**
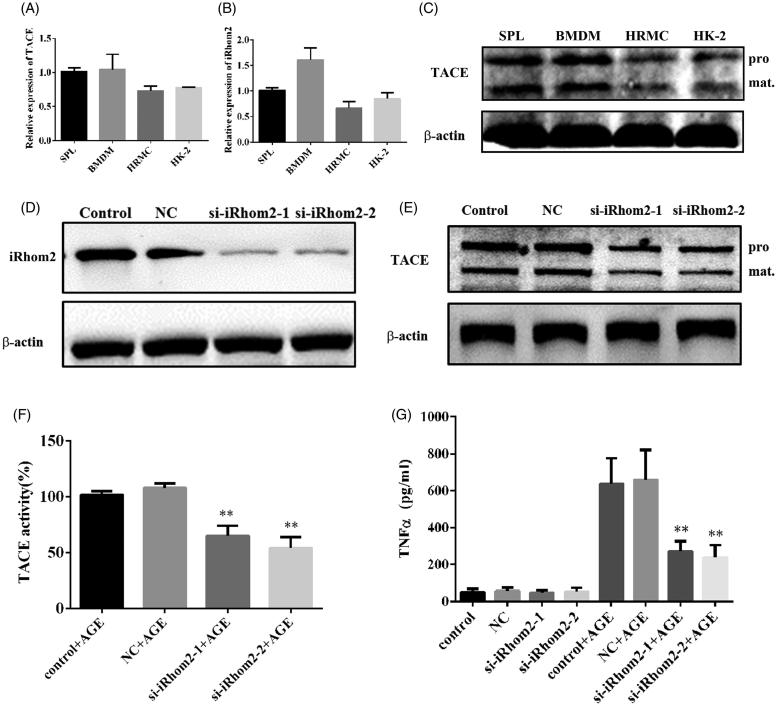
IRhom2/TACE involved in secretion of TNFα in kidney intrinsic cells. (A) The expression of TACE in HRMC, HK-2, BMDM and splenocytes analysed by real-time PCR; (B) the expression of iRhom2 in HRMC, HK-2, BMDM and splenocytes analysed by real-time PCR; (C) western blotting analysis of the expression of TACE in HRMC, HK-2, BMDM and splenocytes, pro: pro-TACE, mat: mature TACE; (D) western blotting analysis knockdown efficiency of two siRNA of iRhom2 in HK-2 cells; (E) western blotting analysis protein level of TACE in HK-2 cells when iRhom2 was knockdown, pro: pro-TACE, mat: mature TACE; (F) the activity of TACE in HK-2 was measured when iRhom2 was knockdown, **p < 0.01, *n* = 3, compared with control; (G) the secretion of TNFα in HK-2 was inhibited when iRhom2 was knockdown by siRNA, **p < 0.01, *n* = 3, compared with control.

TNFα is the primary trigger of inflammation which is involved in the development and progression of DN. It has been reported TNFα signalling requires iRhom2 to promote trafficking and activation of TACE in mammalian cells such as macrophage. To investigate whether iRhom2/TACE involved in kidney intrinsic cells secretion of TNFα, we analysed the expression of iRhom2/TACE in HRMC and HK-2 cell lines by real-time PCR. The results showed iRhom2 and TACE expressed both in HRMC and HK-2 cell lines, even though the expression levels were lower than in bone marrow derived macrophage cells (BMDMs) and splenocytes (Figure 1(A,B)). The western blotting confirmed the protein level of TACE in HRMC and HK-2 (Figure 1(C)). Additional, the protein level of TACE was downregulated (Figure 1(E)) and the activity of TACE induced by AGE in HK-2 was inhibited when iRhom2 was knockdown by siRNAs (Figure 1(D,F)), resulting in inhibiting the secretion of TNFα (Figure 1(G)). Our results show iRhom2/TACE signalling involved in kidney intrinsic cells secretion of TNFα.

### Effects of TFA on AGEs-induced ER-stress and activation of iRhom2/TACE signalling in HK-2 tubule epithelial cell

**Figure 2.**
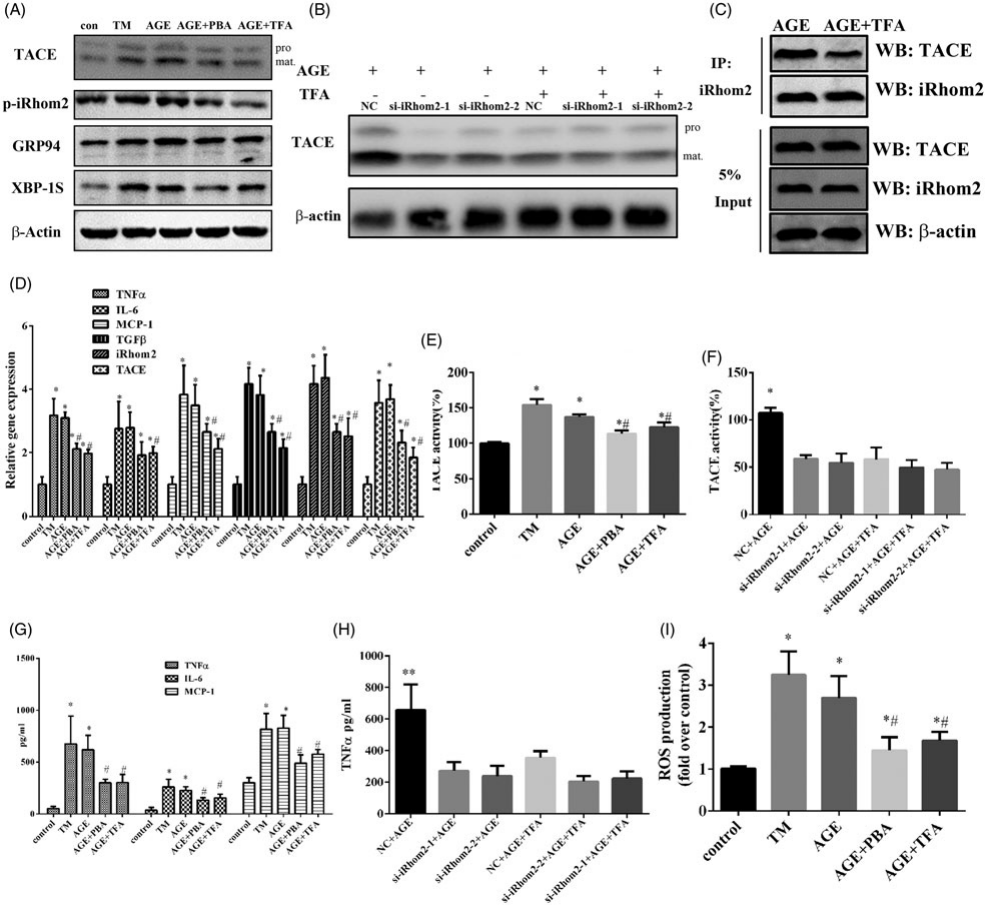
TFA attenuates ER-stress and inhibits activation of iRhom2/TACE signalling induced by AGEs in HK-2 tubule epithelial cell. (A) Western blot analysis of the activation of iRhom2/TACE signalling and the ER stress markers XBP1S and GPR94 are shown, pro: pro-TACE, mat: mature TACE; (B) western blotting analysis protein level of TACE in HK-2 cells induced by AGEs when iRhom2 was knockdown treated with TFA or not, pro: pro-TACE, mat: mature TACE; (C) immunoprecipitation analysis of iRhom2 binds to TACE when treated with TFA; (D) real time PCR showed that TFA decreased expression of iRhom2, TACE and inflammatory cytokine genes in HK-2 induced by AGEs.*p < 0.05 vs. control, ^#^*p* < 0.05 vs. AGE, *n* = 3; (E) TFA inhibited the activity of TACE in HK-2 induced by AGEs, *p < 0.05 vs. control, ^#^*p* < 0.05 vs. AGE, *n* = 3; (F) both iRhom2 knockdown and TFA inhibited the activity of TACE in HK-2 induced by AGEs, *p < 0.05 vs. control, ^#^*p* < 0.05 vs. AGE, *n* = 3; (G) measurement of the secretion of inflammatory cytokines in HK-2 by ELISA, *p < 0.05 vs. control, ^#^*p* < 0.05 vs. AGE, *n* = 3; (H) measurement of the secretion of TNF-α in HK-2 by ELISA when iRhom2 was knockdown treated with TFA or not, **p < 0.01 vs. the other groups, *n* = 3; (I) TFA decreased ROS production in HK-2 induced by AGEs, *p < 0.05 vs. control, ^#^*p* < 0.05 vs. AGE, *n* = 3.

Given the role of iRhom2/TACE signalling in TNFα secretion, iRhom2 may represent an attractive therapeutic target in DN. Total flavone of *Abelmoschus manihot* (TFA) has been proved with the effect on ameliorating renal inflammation but the molecular mechanism is not clear. As TNFα is the primary trigger of inflammation, it was supposed that TFA may inhibit the activation of iRhom2/TACE signalling. With this purpose, we examined the effects of TFA on AGEs-induced ER-stress and activation of iRhom2/TACE signalling in HK-2 tubule epithelial cell. As shown in Figure 2(A), AGEs could induce ER-stress in HK-2 as well as tunicamycin which is a positive drug inducing ER-stress (Garcia-Marques et al. 2015). However, AGEs induced ER-stress was attenuated by TFA or 4-PBA which is an inhibitor of ER-stress (Figure 2(A)) as the ER-stress related proteins GPR94 and XBP1S were downregulated. Even more, TFA or PBA treatment also inhibiting the expression of iRhom2 (Figure 2(A)) and ameliorating iRhom2 binds to TACE (Figure 2(C)), resulting in the mature of TACE was suppressed (Figure 2(A)).

Furthermore, the activity of TACE was also decreased by PBA or TFA treatment (Figure 2(E)). The expression and secretion of inflammatory factors such as TNFα, IL-6 and MCP-1 were also inhibited by TFA or PBA treatment (Figure 2(D,G)). We further studied whether TFA treatment inhibited the secretion of inflammatory factors mainly through iRhom2/TACE signalling. The results showed both TFA treatment and iRhom2 knockdown in HK2 cells inhibited the mature and the activity of TACE induced by AGEs (Figure 2(B,F)). Additionally, TFA treatment as well as iRhom2 knockdown inhibited the secretion of TNF-α in HK2 cells; however, TFA could not further weaken the secretion of TNF-α in HK2 significantly after iRhom2 was knockdown (Figure 2(H)). All of the above results show that anti-inflammatory effect of TFA mainly through inhibiting the activation of iRhom2/TACE signalling in HK2 cells. AGEs induced injury of HK-2 was estimated by the expression of TGFβ1 and the production of ROS, both of them were downregulated by PBA or TFA treatment (Figure 2(D,I)). All of above results indicate TFA with the effects on ameliorating AGE-Induced ER-stress and inhibiting activation of iRhom2/TACE signalling in HK-2 tubule epithelial cell. Due to inhibiting activation of iRhom2/TACE signalling, secretion of TNFα in kidney intrinsic cells induced by AGEs makes TFA as a possible DN treatment agent.

### TFA dose-dependently improved renal injury in DN rats

A streptozotocin-induced rat model of DN was used to examine the *in vivo* protective effects of TFA on DN. DN rats were treated orally every day from 8 to 20 weeks of age with low, middle and high doses of TFA (low dose: 75 mg/kg; middle dose: 135 mg/kg; high dose: 300 mg/kg). PBA (2.5 mg/kg) was used as positive treatment. The DN rats showed listlessness, low activity, loss of appetite, mild diarrhoea, weight loss, much water intake and urine output. Treatment with TFA and PBA both improved all of the disorders in the DN rats (Table 1), and the effects were dose-dependent. The BW of HD-TFA group was increased significantly in contrast to the DN rats without treat or LD-TFA group at the end of the 12th week, even more, the ratio of KW and BW was decreased statistically significantly after treatment with HD-TFA (HD-TFA group vs. DN group). No obvious difference in the ratio of KW/BW was observed between the DN group and LD-TFA group. Meanwhile, the effects of TFA on blood biochemical parameters were investigated. Compared with the DN group, Scr and BUN in PBA, HD-TFA, MD-TFA and LD-TFA groups were decreased after the administration of drugs (Table 1). However, the differences of Scr and BUN among DN, PBA, HD-TFA MD-TFA and LD-TFA groups were not statistically significant. In contrast with the non-DN group, a significant increase of serum Alb in the HD-TFA group was observed compared with DN and LD-TFA groups. These results demonstrate that TFA improves metabolic disorders in DN rats.

**Table 1.** Physical and biochemical parameters in various groups of rats at 12 weeks.

	Non-DN	DN + vehicle	DN + LD-TFA	DN + MD-TFA	DN + HD-TFA	PBA
BW (g)	526 ± 31	241 ± 33^a^	276 ± 44^a^	325 ± 56^a^	393 ± 28^a,b^	399 ± 29^a,b^
KW/BW (g/kg)	2.94 ± 0.15	6.75 ± 0.73^a^	6.11 ± 0.71^a^	5.61 ± 0.42^a^	5.31 ± 0.41^a^	5.64 ± 0.65^a^
Urine (mL/24 h)	30 ± 12	281 ± 43^a^	259 ± 32^a^	218 ± 27^a^	182 ± 29^a,b^	191 ± 36^a,b^
Water (mL/24 h)	47 ± 13	3378 ± 42^a^	329 ± 49^a^	291 ± 47^a^	261 ± 45^a^	265 ± 47^a^
Food (g/24 h)	34 ± 6	19 ± 3^a^	22 ± 7^a^	26 ± 6	32 ± 5	30 ± 5
Upro (mg/24 h)	11.15 ± 3.60	56.35 ± 6.12^a^	54.15 ± 7.10^a^	33.35 ± 6.52^a^	25.35 ± 5.71^a,b^	26.15 ± 3.80^a,b^
UAE (mg/24 h)	1.60 ± 0.51	21.09 ± 11.18^a^	19.14 ± 6.12^a^	13.57 ± 3.13^a^	8.45 ± 3.16^a,b^	7.12 ± 3.62^a,b^
BUN (mmol/L)	5.74 ± 0.73	11.32 ± 2.16^a^	10.23 ± 1.18^a^	9.18 ± 2.03^a^	8.56 ± 1.67^a^	7.12 ± 2.21^a^
Scr (mmol/L)	28.32 ± 2.78	43.02 ± 4.67^a^	41.12 ± 4.12^a^	39.86 ± 4.05^a^	37.12 ± 4.30^a^	35.13 ± 3.87^a^
Alb (g/L)	32.17 ± 4.89	21.47 ± 3.78^a^	24.68 ± 4.77^a^	26.15 ± 4.13	29.10 ± 3.13^b^	28.92 ± 3.31^b^
BG (mmol/l)	5.48 ± 1.95	26.24 ± 3.35^a^	25.12 ± 2.76^a^	22.23 ± 4.18^a^	18.11 ± 2.76^a,b^	19.13 ± 3.21^a,b^

BW: body weight; KW: kidney weight; Upro: urinary protein; UAE: urinary albumin excretion; BUN: serum blood urea nitrogen; Scr: serum creatinine; Alb: serum albumin; BG: blood glucose.

The data are expressed as means ± SD.

a *p* < 0.05 vs. non-diabetic rats.

^b^*p* < 0.05 vs. untreated DN rats.

**Figure 3.**
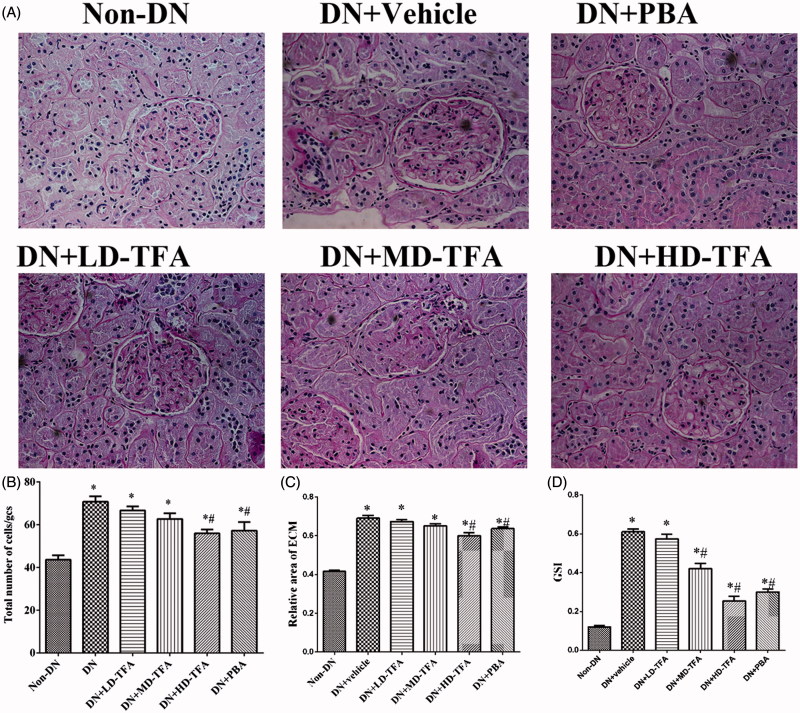
Effects of TFA on the formation of glomerulosclerosis fibrosis in the renal tissues of STZ-induced DN rats. (A) Representative periodic acid-Schiff staining of paraformaldehyde-fixed kidney sections from the rats of each group (*n* = 10 rats/group): non-DN, DN + vehicle, DN + PBA, DN + low dose (LD)-TFA, DN + middle dose (MD)-TFA and DN + high dose (HD)-TFA; (B) glomerular changes were evaluated by total number of cells per glomerular cross-section (GCS); (C) glomerular injury was detected by relative area of extracellular matrix (ECM), magnification, 400×. The data are expressed as mean ± SD. *p < 0.05 vs. non-DN, ^#^*p* < 0.05 vs. DN + vehicle; (D) semi-quantitative analyses of glomerulosclerosis index (GSI) in DN rats with or without TFA treatment, the data are expressed as mean ± SD. *p < 0.05 vs. non-DN, ^#^*p* < 0.05 vs. DN + vehicle.

Additionally, we compared levels of urinary protein among all of experimental groups. With the administration of drugs, the urinary protein of DN and LD-TFA groups declined slowly, but both of HD-TFA and PBA groups were reduced significantly at the end of the treatment (Table 1). PAS staining showed mesangial tissues of DN rats were under hyperplasia, including glomerular mild hypertrophy, capillary loop area reduction, MC proliferation, ECM expansion, and diffused increasing areas of ECM (Figure 3(A)). Marked expansion of ECM in DN rats was greatly improved by TFA treatment (Figure 3(C)). Treatment with HD-TFA or PBA attenuated the injurious glomerular morphological changes in DN rats, which showed better improvements than the LD-TFA treatment (Figure 3(A)). With treatment of TFA at a high dose, the scores were alleviated in the DN group (total number of cell in 29 glomerular cross-sections: HD-TFA group 57.37 ± 6.19 vs. DN group 71.11 ± 6.83; relative area of ECM: HD-TFA group 0.63 ± 0.04 vs. DN group 0.68 ± 0.06, *p* < 0.05). No significant difference was found between the LD-TFA group and DN group (Figure 3(B,C)).

**Figure 4.**
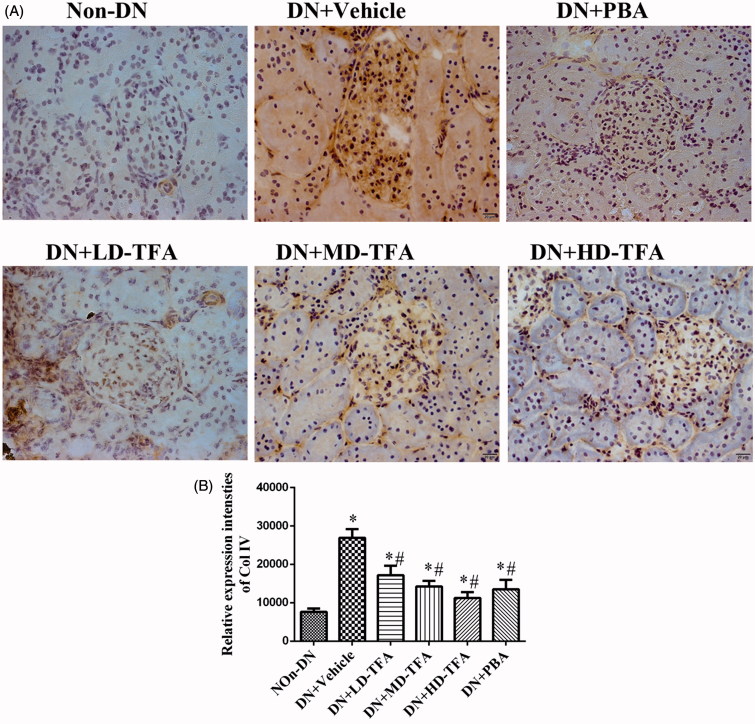
Immunohistochemical staining of collagen type IV in renal tissues. (A) Paraformaldehyde-fixed kidney sections taken from representative rats from each group (*n* = 10 rats/group) at 12 weeks were stained with anti-Col IV polyclonal antibody. Representative images (400×) of kidney samples from the following groups: non-DN, DN + vehicle, DN + PBA, DN + low dose (LD)-TFA, DN + middle dose (MD)-TFA and DN + high dose (HD)-TFA; (B) densitometric analysis of the immunohistochemical staining of renal Col IV protein in 10 rats from each group, *p < 0.05 vs. non-DN, ^#^*p* < 0.05 vs. DN + vehicle.

**Figure 5.**
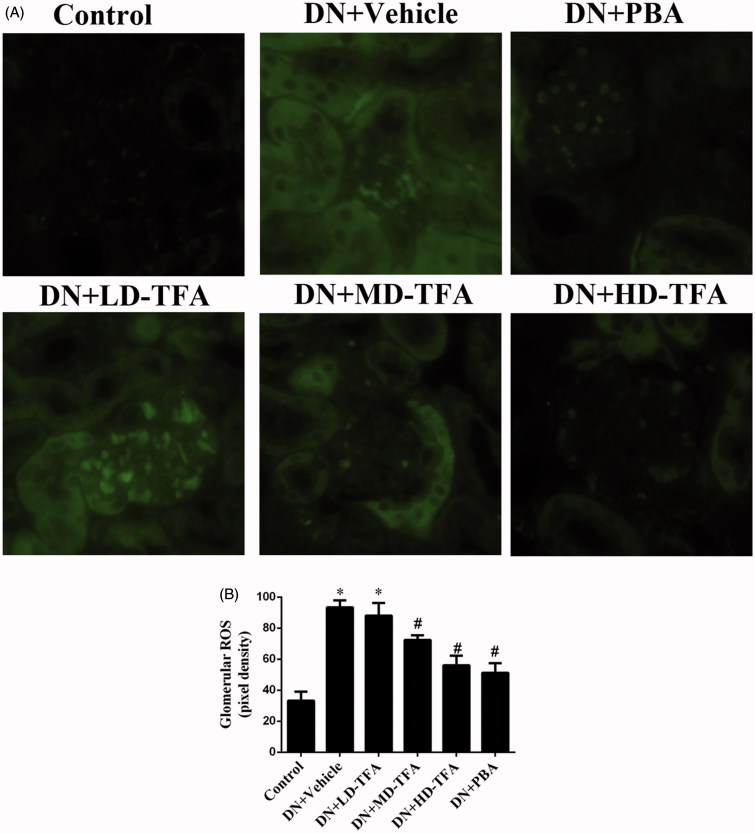
Effect of TFA on kidney ROS production. (A) Representative 2′,7′-dichlorofluorescein (DCF) staining of hydrogen peroxide in the kidney (400×). (B) Quantification of the pixel density of DCF staining in glomeruli. The data are expressed as mean ± SD. *p < 0.05 vs. non-DN, ^#^*p* < 0.05 vs. DN + vehicle, *n* = 10.

Renal injury was also estimated by immunohistochemical staining of collagen type IV and 2′,7′-DCF staining of hydrogen peroxide. Collagen type IV staining (Figure 4(A)) and the generation of ROS in renal glomeruli and proximal tubular epithelial cells (Figure 5(A)) were enhanced in the DN group compared with the non-DN group. Upon treatment of the DN rats with TFA at a high dose, the intense staining of collagen type IV (Figure 4(A)) was attenuated. HD-TFA as well as PBA attenuated expressions of collagen type IV in kidneys of DN rats more significant than LD-TFA. Even more, glomerular ROS generation was decreased by PBA and TFA treatment (Figure 5(A)). TFA attenuated ROS generation in renal glomeruli and proximal tubular epithelial cells in a dose-dependent manner, HD-TFA treatment reduced ROS production in kidney sections obviously while LD-TFA groups without significant differences compared with vehicle treatment (Figure 5). We finally investigated the GSI in DN rats with or without TFA treatment. Semi-quantitative analyses showed that the diabetic rats were with remarkably increased GSI (non-DN, 0.12 ± 0.03 arbitrary units (AU); DN, 0.68 ± 0.06 AU, *p* < 0.05). Strikingly, TFA improved GSI in a dose-dependent manner (LD-TFA, GSI, 0.62 ± 0.04 AU; MD-TFA, GSI, 0.42 ± 0.03 AU; HD-TFA, GSI, 0.26 ± 0.04 AU, *p* < 0.05) (Figure 3(D)).

The results shown above indicate that TFA could ameliorate proteinuria, Alb, and glomerular morphological changes, and attenuate the expressions of collagen type IV and ROS generation in glomeruli in DN rats in a dose-dependent manner.

### Effects of TFA in activation of iRhom2/TACE signalling and inflammatory processes in DN rats

**Figure 6.**
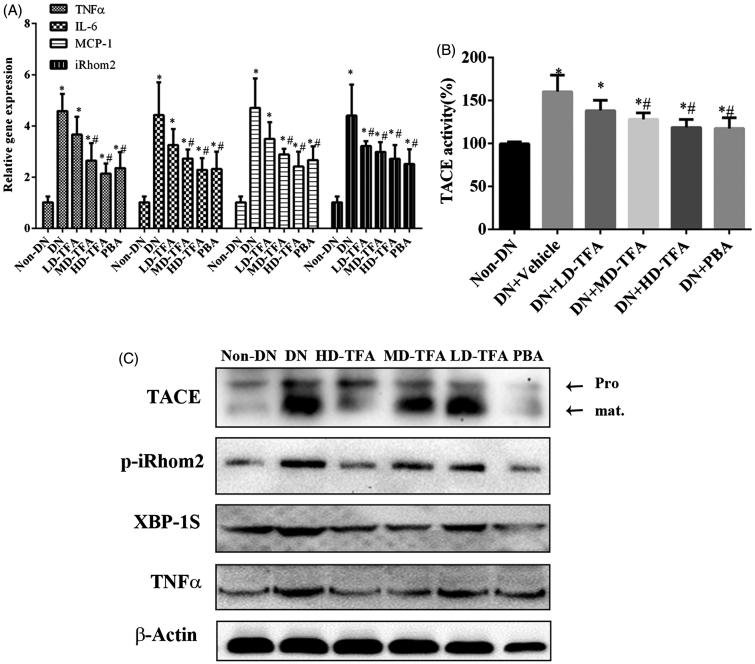
TFA attenuates inflammatory processes and inhibits activation of iRhom2/TACE signalling in DN rats. (A) TFA decreased expression of inflammatory cytokine genes in renal tissues of DN rats, *p < 0.05 vs. non-DN, ^#^*p* < 0.05 vs. DN + vehicle, *n* = 10; (B) TFA inhibited the activity of TACE in renal tissues of DN rats, *p < 0.05 vs. non-DN, ^#^*p* < 0.05 vs. DN + vehicle, *n* = 10; (C) western blotting analysis of the activation of iRhom2/TACE signalling, the activation of TNFα and the expression of ER stress markers XBP1S and GPR94 in renal tissues of DN rats.

We next explored whether the molecular mechanisms of TFA improve DN correlation with iRhom2/TACE signalling. We first examined the expression of iRhom2 and TACE mRNA in freshly isolated tissue of kidney by quantitative real-time PCR. As shown in Figure 6, TFA administration decreased expression of iRhom2 mRNA in kidneys of DN rats. The decreased expression of iRhom2 in kidney after TFA treatment was confirmed by western blotting. Although TFA or PBA administration did not affect the expression of TACE mRNA in kidney of DN rats significantly, western blotting showed the mature TACE was repressed in HD-TFA and PBA groups compared with DN group. As the mature TACE affords its activity, we measured the activity of TACE in kidney of DN rats treated with or without TFA. From Figure 6(B), it could be found both TFA and PBA treatment inhibited the activity of TACE. Moreover, treatment with PBA which is a specific inhibitor of ER-stress also inhibited the expression of iRhom2 in kidney of DN rats (Figure 6(A)). TFA as well as PBA treatment decreased the expression of ER-stress related protein XBP1S which suggested TFA with the effect on ameliorating ER-stress in kidney of DN rats. According to the above results, the effects of TFA on inhibiting iRhom2/TACE signalling in kidney of DN rats perhaps through ameliorating ER-stress more or less.

Based on our *in vitro* data suggesting that TFA regulates AGE induced activation of TNFα by iRhom2/TACE signalling, we next examined whether inhibition of iRhom2/TACE signalling by TFA treatment could affect TNFα levels during DN. We examined the expression profile of proinflammatory cytokines in the renal tissues of TFA-treated DN rats. The results show the vehicle treatment DN group with elevated levels of TNFα, IL-6, IL-1 and IL-2 compared with the non-DN group, treatment with HD-TFA significantly reduced the expression of these cytokines (Figure 6(A)). TFA treatment could inhibit the activation of iRhom2/TACE signalling in kidney of DN rats suggesting the secretion of TNFα might be inhibited as TNF secretion in mammalian cells depends on the activity of iRhom2/TACE signalling. Further, we measured the activation and secretion of TNFα by ELISA and western blotting. Experimental results showed treatment with TFA dose-dependently decreased the activation and secretion of TNFα in the DN rats (Figure 6(C,D)). As TNFα is the primary trigger of inflammation, the effects of TFA on ameliorating renal inflammation are perhaps by way of inhibiting the activity of iRhom2/TACE signalling. These results show that TFA could downregulate the protein expression of proinflammatory cytokines in the kidneys of DN rats and exert anti-inflammatory effects through inhibiting the activation of iRhom2/TACE signalling in kidney of DN rats.

## Discussion

TFA represents the major active component that has been isolated from the traditional Chinese herb (Liu et al. 2009). Diverse *in vitro* and *in vivo* studies have shown that TFA can be successfully used to treat ischemic/reperfusion injuries and inflammatory diseases (Zhu et al. 2011). Even more, TFA has been reported to reduce renal inflammation and glomerular injury in chronic kidney disease (CKD). However, the exact mechanisms by which TFA elicits its anti-inflammatory effects remain incompletely understood. The cytokine TNF is the primary trigger of inflammation, and TNF signalling requires iRhom2 to promote trafficking and activation of TACE. Therefore, we hypothesized that the anti-inflammatory effects of TFA may act by inhibiting the activation of iRhom2/TACE signalling.

TNFα is a central mediator of inflammatory responses. It is synthesized in a pro-form that must be processed to an active form for secretion and binding to the TNF receptor. TACE is a zinc-dependent membrane-bound disintegrin metalloproteinase responsible for TNFα cleavage and secretion (Christova et al. 2013). Its activity is regulated by iRhoms. Although iRhom2 signalling is widely present in monocytes such as macrophage, whether iRhom2 signalling regulates the secretion of TNFα in kidney intrinsic cells was not known. Our results show considerable expression of iRhom2 and TACE in HRMC and HK-2 cell lines (Figure 1), even though the expression level was significantly lower than in macrophages and splenocytes. The activity of TACE was inhibited when iRhom2 was knocked down by siRNA in kidney intrinsic cell lines (Figure 1). Knockdown of iRhom2 also inhibited the secretion of TNFα. These results suggest that iRhom2/TACE signalling plays a key role in the secretion of TNFα in the kidney.

Inflammatory cytokines that are involved in the pathogenesis of diabetes play a significant role in the development and progression of several renal disorders including DN (Noronha et al. 1995; Mora and Navarro 2004). Therefore, anti-inflammatory strategies may offer approaches of great interest in treating DN. In our study, we characterized the effects of TFA as a possible natural inhibitor of iRhom2/TACE signalling. We found that an increase in TNF-α shedding in cells exposed to AGEs (Figure 2) was associated with a corresponding increase in the maturation of TACE (Figure 2). Tunicamycin, an inducer of ER stress (Garcia-Marques et al. 2015; Xing et al. 2016) also increased the activation of TACE and TNFα shedding. TFA or PBA treatment inhibited the ER stress and the activation of iRhom2/TACE signalling that was induced by AGE *in vitro* (Figure 2). The ability of TFA to inhibit the activation of iRhom2/TACE signalling that is induced by AGE makes it a possible therapeutic agent in the treatment of DN.

The protective effects of TFA on DN were also examined *in vivo*. We found that TFA significantly reduced the expression of inflammatory factors (Figure 6(A)), reduced progressive renal damage and inhibited glomerulosclerosis fibrosis (Figures 3 and 4). Even more, TFA exerted beneficial effects on metabolic disorders and glucose metabolism in DN rats in a dose-dependent manner (Table 1). These effects suggest that TFA could provide a suitable therapeutic approach for the treatment of DN. The possible molecular mechanisms by which TFA improves renal damage were also investigated. TFA downregulated expression of iRhom2 and inhibited the activity of TACE, reducing the levels of TNFα (Figure 6). Other proinflammatory factors such as IL6 and MCP-1 were also reduced by TFA treatment (Figure 6). Additionally, ROS generation in renal tissues of DN rats was also reduced by TFA (Figure 5). These effects may all contribute to reduced renal damage in DN rats.

Although the mechanisms by which TFA inhibits the activation of iRhom2/TACE signalling that is induced by AGE remain unclear, our studies suggest that the beneficial inhibition of iRhom2/TACE signalling by TFA could be attributable, at least in part, to its ability to alleviate ER stress. As the ER is a central organelle involved in lipid synthesis, protein folding, protein maturation, quality control and trafficking (Dadras and Khoshjou 2015), ER stress is receiving greater attention as a cause of DN. Accumulating evidence suggests that ER stress plays an essential role in the development and pathogenesis of DN. Therefore, agents that alleviate ER stress may act as potent anti-DN agents and could have clinical application for the treatment of DN. In our study, we found both *in vitro* (Figure 2) and *in vivo* (Figure 6) that PBA, an inhibitor of ER stress, downregulated the expression of iRhiom2 and inhibited the activation of TACE that is induced by high glucose. XBP1S, which is an indicator of ER stress, was higher in HRMC or HK-2 cells that were exposed to AGEs and in the kidneys of DN rats. Interestingly, the level of XBP1S markedly decreased with TFA treatment (Figure 6(C)). All of these results demonstrate direct protective effects of TFA against ER stress. However, the mechanisms by which TFA controls ER stress and whether this is the only physiological target of TFA’s protective effects needs further research.

## Conclusions

Our results revealed that TFA, the major active component isolated from the traditional Chinese herb *Abelmoschus manihot* medic, ameliorates renal inflammation and glomerular injury in DN rats by alleviating ER stress and repressing the activity of iRhom2 signalling that is induced by AGEs. However, it must be emphasized that TFA is a mixture extracted from AM, so further research is needed to reveal the molecular mechanisms by which TFA inhibits the activation of iRhom2/TACE signalling in kidneys that is induced by AGEs.
